# Antimalarial Activity of the Leaf Latex of *Aloe weloensis* (Aloaceae) against *Plasmodium berghei* in Mice

**DOI:** 10.1155/2020/1397043

**Published:** 2020-08-14

**Authors:** Tekleab Teka, Tadesse Awgichew, Haile Kassahun

**Affiliations:** Department of Pharmacy, College of Medicine and Health Sciences, Wollo University, Dessie, Ethiopia

## Abstract

**Background:**

Emergence of drug resistance and lack of therapeutic efficacy of modern antimalarial drugs are the most triggering factors for the searching of new lead compounds with different mechanisms of action. Medicinal plants with documented traditional uses are a viable option for treatment of malaria. Traditionally, the leaf latex of *Aloe weloensis* has been used in the treatment of malaria in Ethiopia. Hence, this study was undertaken to investigate the antimalarial activity of the leaf latex of *Aloe weloensis* in *Plasmodium berghei*-infected mice.

**Methods:**

A four-day suppressive test was employed to evaluate the antimalarial effect of the leaf latex of the plant against *P*. *berghei* in Swiss albino mice. Mice were randomly assigned in five groups of five animals in each and given 100, 200, and 400 mg/kg of the leaf latex, chloroquine 25 mg/kg, and distilled water. The level of parasitemia, packed cell volume, survival time, temperature, and body weight was used to determine the antimalarial activity.

**Results:**

The acute toxicity study indicated that the leaf latex of *A*. *weloensis* caused neither mortality nor signs and symptoms of toxicity at a dose of 2000 mg/kg. Furthermore, the 4-day suppressive test indicated that the latex of *the plant* exhibited a significant parasitemia reduction in a dose-dependent manner as compared to negative control. The leaf latex of the plant exhibited a percent inhibition of 13.05%, 41.87%, and 66.84% at doses of 100 mg/kg, 200 mg/kg, and 400 mg/kg, respectively. The chemosuppression of the antimalarial activity was statistically significant at 100 mg/kg (*p* < 0.05), 200 mg/kg (*p* < 0.01), and 400 mg/kg (*p* < 0.01) as compared to negative control. All doses of the leaf latex prevented weight loss and reduction in temperature and packed cell volume and increased the survival time of infected mice.

**Conclusion:**

The results of this study demonstrated that the leaf latex of *Aloe weloensis* possessed antiplasmodial activity confirming the genuine traditional use of the plant as an antimalarial agent.

## 1. Background

Malaria is a major infectious disease caused by protozoan parasites from the genus *Plasmodium* [1]. The sub-Saharan African region has the highest risk of malaria infection. Children under five years of age and pregnant women are most severely affected [2]. Malaria is ranked as the leading communicable disease in Ethiopia [3].

The alarming rate at which malaria parasites develop resistance to most of the available and affordable antimalarial drugs is a major concern that urgently requires the development of newer and more effective alternatives [4, 5]. The development of resistance to the antimalarial drug such as chloroquine and sulfadoxine-pyrimethamine has redirected treatment strategies to artemisinin-based combination therapies, which are now WHO-recommended treatment regimens [6]. Despite several actions and strategies in public health employed for control and eradication of malaria, the goal has still not been achieved and malaria continues to have a strong impact on the health [7]. In Ethiopia, 7.5% treatment failure on the artemisinin-based combination regimen was currently reported [8]. To date, there are no new medicines in advanced stages of development to replace the artemisinin, and this urges the finding of new antimalarial compounds from plants.

Traditionally used herbal medicines have been behind many drug discovery successes including antimalaria agents. Antimalarial drugs such as artemisinin and quinine are natural products derived from traditional herbal remedies that are used to treat fevers [9]. In the sub-Saharan African region, including Ethiopia, hundreds of plants are traditionally used for the treatment of malaria [10]. Nowadays, several traditionally claimed Ethiopian medicinal plants showed promising antimalarial activity [11].

*A*. *weloensis*, locally known as Eret tafa, belongs to a family of Aloaceae. The leaf latex of *A*. *weloensis* (Eret tafa) is traditionally used for the treatment of malaria, wound, different skin diseases and pain from ear infection, headache, and rheumatism. An ethnobotanical survey conducted in Gubalafto, Northeast Ethiopia, reported that the leaf latex of the plant is taken orally to treat malaria [12, 13]. However; the antimalarial activity of this medicinal plant is not scientifically studied. Hence, the present study was designed to evaluate the antimalarial activity of the leaf latex of *Aloe weloensis* against *plasmodium berghei*-infected mice.

## 2. Materials and Methods

### 2.1. Plant Material

The leaf latex of *A*. *weloensis* was collected from Gubalafto, North Wollo, Northeast Ethiopia, in February, 2019. The leaf latex was kept in plastic containers during transportation. The plant was authenticated by a taxonomist at the National Herbarium, Department of Biology, Addis Ababa University, and was given a voucher specimen TA001 for future use.

### 2.2. Collection of the Latex

The leaf latex of *A. weloensis* was collected by cutting the leaves transversely near the base and allowing the yellow sap to come down in a plastic material. The sap was, then, left in open air for three days to allow evaporation of water, which yielded a pale yellow latex. Finally, after drying, the dried latex was transferred into a vial and, then, kept in a refrigerator until further use [13–15].

### 2.3. Experimental Animals

Healthy Swiss albino mice of either sex with the weight of 20–35 g obtained from the Department of Pharmacy, Wollo University, were used. Animals were kept in a 12-hour light- dark cycle and provided with food and water ad libitum. They were acclimatized for a week before starting the experiment. All experiments were conducted in accordance with the guide for care and use of laboratory animals [16].

### 2.4. Phytochemical Screening

The leaf latex of the plant was screened for the presence of different phytochemical constituents including alkaloids, anthraquinones, glycosides, saponins, terpenoids, tannins, and flavonoids using standard procedures [17, 18].

### 2.5. Acute Toxicity Study

The leaf latex of *A*. *weloensis* was evaluated for acute toxicity according to the OECD guideline [19], and five noninfected female Swiss albino mice were used. They were fasted from food overnight. The leaf latex was administered sequentially at a dose of 2000 mg/kg. If no death was observed in 24 hours, an additional four mice were administered sequentially. The mice were observed for signs of toxicity such as lacrimation, hair erection, loss of appetite, salivation, diarrhea, and mortality over 4 h, 24 h, and for 14 days.

### 2.6. Parasite Infection of Experimental Animals

Chloroquine-sensitive strain of *Plasmodium berghei* (ANKA), obtained from the Ethiopian Public Health Institute (EPHI), was used to infect experimental animals. The parasite was maintained by serial passage of blood from infected mice to noninfected mice weekly [20]. To infect mice, blood from donor mouse with a parasitemia level of 30–37% was collected into heparinized tubes containing 0.5% trisodium citrate. The blood was, then, diluted with normal saline to obtain about 1 × 10^7^ parasitized RBCs in every 0.2 ml suspension [8]. Each experimental animal was infected intraperitoneally with 0.2 ml of infected blood containing 1 × 10^7^ parasitized RBCs.

### 2.7. Dosing and Grouping of Animals

*P*. *berghei*-infected mice were randomly divided to five groups, each consisting of five mice. Group I (negative control) was treated with 10 ml/kg distilled water; group II, III, and IV were treated with three different doses of the leaf latex, 100 mg/kg, 200 mg/kg, and 400 mg/kg, respectively; and group V (positive control) was treated with chloroquine (25 mg/kg) [14].

### 2.8. Four-Day Suppressive Test

Evaluation of the antimalarial activity of the plant was carried based on the methods described by Peter et al. [21]. Treatment was started three hours after the mice were infected with the parasite on the first day (D_0_). Treatment was, then, continued daily for four days (D_0_–D_3_). On the 5th day (D_4_), blood was collected from the tip tail of each mouse, and thin blood films were made. The air-dried thin films were fixed with 100% methanol and stained with 10% Giemsa stain at pH 7.2. Body weight, rectal temperature, % inhibition, parasitemia, and survival time were measured.

### 2.9. Parasitemia and Survival Time Determination

The parasitemia was determined by counting the number of parasitized erythrocytes in random fields of the microscope. % parasitemia and % suppression were calculated by using the following formula, respectively [22]:

(1) $$\begin{array}{c} \text{\% Paracetemia}=\;\frac{\text{Number of Parasitized RBC }}{\text{Total Number of RBC}}\times 100,\;\;\\ \text{\% Suppression}=\frac{\left(\text{Parasitemia of Negative Control}-\text{ Parasitemia of Treated Group}\right)\;}{\text{Paracetemia of Negative Control}}\times 100. \end{array}$$

Mortality was monitored daily, and the number of the days from the time of infection up to death was recorded for each experimental animal throughout the follow-up period. Mean survival time (MST) was calculated for each group using the following formula [23]:

(2) $$\begin{array}{c} \text{MST}=\frac{\text{Sum of Survival Time of all Mice in a Group }\left(\text{Days}\right)}{\text{Total Number of Mice in that Group}}. \end{array}$$

### 2.10. Packed Cell Volume Determination

Heparinized capillary tubes were used for the collection of blood from tail of the mice. The tubes were filled to 3/4^th^ of their height and sealed by a sealant and were placed in a microhematocrit centrifuge. The blood was centrifuged at 12,000 rpm for 15 min, and PCV was determined using a standard microhematocrit reader. PCV of each mouse was measured before infection (D_0_) and after infection (D_4_) as follows [24]:

(3) $$\begin{array}{c} \text{PCV}=\frac{\text{Volume of Erythrocytes in a Given Volume of Blood}}{\text{Total Blood Volume}}. \end{array}$$

### 2.11. Body Weight and Rectal Temperature Determination

The body weight of each mouse was measured before infection (D_0_) and after infection (D_4_) using a sensitive digital analytical balance. Rectal temperature was also measured by using a digital thermometer before infection and after infection [23].

### 2.12. Ethical Clearance

This study was approved by the ethical review committee of Wollo University, College of Medicine and Health Sciences, Ethiopia (protocol number: WU Phar/397/2010). The experiment was performed in accordance with the guide for the care and use of laboratory animals [25].

### 2.13. Statistical Analysis

The data were expressed as mean ± standard error of the mean. Data were analyzed using SPSS version 23. Statistical significance was determined by one-way ANOVA followed by the Tukey post hoc test to compare the levels of parasitemia, survival time, and changes in body weight, PCV, and rectal temperatures between control and latex-treated groups. A *p* value of <0.05 was considered statistically significant.

## 3. Results

### 3.1. Preliminary Phytochemical Screening

Preliminary phytochemical screening of the leaf latex of the plant revealed the presence of anthraquinones, glycosides, saponins, terpenoids, tannins, and flavonoids while alkaloids were absent (Table 1).

### 3.2. Acute Toxicity Study

The acute toxicity study indicated that the leaf latex of *A*. *weloensis* did not cause mortality of mice within 24 hours of treatment, as well as during 14 days. Gross physical observation of mice revealed no visible signs of acute toxicity such as lacrimation, hair erection, loss of appetite, salivation, and diarrhea.

### 3.3. Four-Day Suppressive Test

The 4-day suppressive test indicated that the leaf latex of *A*. *weloensis* exhibited a significant parasitemia reduction in a dose-dependent manner as compared to negative control. The leaf latex exhibited a percent inhibition of 13.05%, 41.87%, and 66.84% at doses of 100 mg/kg, 200 mg/kg, and 400 mg/kg, respectively. The chemosuppression of the antimalarial activity was statistically significant at 100 mg/kg (*p* < 0.05), 200 mg/kg (*p* < 0.01), and 400 mg/kg (*p* < 0.01) as compared to negative control. Chloroquine had a chemosuppression of 100% at the dose level of 25 mg/kg/day and showed highly significant (*p* < 0.01) suppression as compared to latex-treated and negative control groups (Table 2).

All doses of the leaf latex exhibited a dose-dependent significant increment of MST compared to negative control. 200 mg/kg and 400 mg/kg doses of the leaf latex were able to significantly (*p* < 0.01) prolong the survival time as compared to negative control. All mice treated with chloroquine, 25 mg/kg/day, survived throughout the monitoring period (>25 days), as shown in Table 2.

All doses of the leaf latex prevented body weight loss in a dose-dependent manner as compared to the negative control. 100 mg/kg (*p* < 0.05), 200 mg/kg (*p* < 0.05), and 400 mg/kg (*p* < 0.01) of the leaf latex prevented body weight reduction significantly as compared to negative control (Table 3).

As shown in Table 3, different doses of the leaf latex of *A*. *weloensis* prevented reduction of temperature in a dose-dependent manner. 400 mg/kg (*p* < 0.01) doses of the leaf latex prevented the decrease in rectal temperature significantly as compared to water-treated groups.

The leaf latex of *A*. *weloensis* significantly prevented reduction of PCV as compared to the negative control. The latex was able to significantly (*p* < 0.01) prevent PCV reduction at 200 mg/kg and 400 mg/kg doses as compared to water-treated groups (Table 3).

## 4. Discussion

Many antimalarial drugs currently available on the market have been developed from plants and natural products. Antimalarial drug resistance remains a major challenge and continues to emerge creating an obstacle in malaria control and elimination. *Plasmodium falciparum* resistance to the existing antimalarials necessitates the development of improved drug interventions. At present, developing novel approaches and new alternative antimalarial drugs is pivotal to combat the disease [26, 27].

The present study investigated the phytochemical contents, acute toxicity, and antimalarial activity of the leaf latex of *A*. *weloensis* against *P*. *berghei*-infected mice. A phytochemical study of the leaf latex indicated the presence of anthraquinones, glycosides, saponins, terpenoids, tannins, and flavonoids. Phytochemical screening studies on different species of the Aloe genus have also reported the presence of terpenoids, alkaloids, and flavonoids [14].

An acute toxicity study of the plant was carried out in Swiss albino mice prior to the antimalarial activity test. The acute toxicity study indicated that the leaf latex of *A*. *weloensis* caused no mortality at the dose used (2 g/kg) within the first 24 hours and for the next 14 days. Additionally, no major signs of toxicity such as changes in general behavior, variations in body weight, and mortality were observed. This may suggest the safety of the studied pant and, hence, signifies that the LD_50_ might be greater than 2000 mg/kg.

The four-day chemosuppressive test is a standard test commonly used for antimalarial screening and determination of percent inhibition of parasitemia is the most reliable parameter [16, 28]. In the present study, the leaf latex of *A*. *weloensis* has shown significant antimalarial activity as compared to the negative control in a dose-dependent manner. The highest suppression was observed at the maximum dose given (400 mg/kg). This might be due to phytochemical constituents responsible for the antimalarial activity may present in low levels [29]. Hence, the present study provides the scientific evidence for the folkloric use of the plant in the treatment of malaria. The antimalarial activity of the latex of *A*. *weloensis* could be due to a single compound or synergetic effect of the secondary metabolites found in the plant. Based on the qualitative screening test, the leaf latex of *A*. *weloensis* was found to be positive for the presence of anthraquinones, glycosides, saponins, terpenoids, tannins, and flavonoids, which have been considered to have antimalarial activity [3, 5, 8, 22, 27, 30–32]. Moreover, flavonoids which have antioxidant activity may also contribute to the antimalarial activity. Antioxidant compounds can inhibit hemozoin formation, and free heme is very toxic for malaria parasite [33]. In addition, secondary metabolites such as glycosides have been shown to possess direct antiplasmodial effects [5]. Similar results were obtained in studies reported from Aloe species such as *A*. *pulcherrima* [14], *A*. *megalacantha* [23], *A*. *percrassa* [15], *Aloe debrana* [6], *A*. *citrana* [34], *and A*. *macrocarpa* [11] which have been reported to have significant antimalarial activities.

Survival time is another parameter to evaluate the antimalarial activity of plant extracts, and if an extract results in a survival time longer than the that of negative control groups, it is considered as an active agent against malaria [35, 36]. In this study, the leaf latex of the plant significantly (*p* < 0.01) improved the survival time of *P*. *berghei*-infected mice at 200 mg/kg and 400 mg/kg doses relative to the negative control which confirms that the latex of the plant contain antimalarial compounds which reduce the number of parasites and, hence, prolongs the survival time. *A*. *pulcherrima* [14], *A*. *pirottae* [1], and *A*. *megalacantha* [23] have also shown similar results. In the present study, there has been a strong association between the mean survival time and the suppression capacity of the plant. This finding might indicate that the latex suppressed *P*. *berghei* and reduced the overall pathologic effect of the parasite in the infected mice.

Body weight loss, reduction in PCV, and low body temperature are cardinal signs of malaria-infected mice. Hence, ideal plant extracts with antimalarial activity are expected to prevent malaria-associated reduction of body weight, PCV, and temperature due to the rise in parasitemia [9].

Weight loss is a characteristic of malaria infection resulted from appetite loss, metabolic disturbance, and hypoglycemic effect of the parasite [35]. Body weight loss prevention is also another parameter to confirm the antimalarial activity of new natural or synthetic antimalarial drugs. Antimalarial agents obtained from plants are expected to prevent body weight loss in infected mice due to an overall pathologic effect caused by the parasite [36]. In this study, the latex of *A*. *weloensis* significantly prevented weight loss at all dose levels compared to the negative controls. Thus, prevention of weight loss after treatment with the leaf latex could be due to suppression of the parasite and/or enhanced appetite among treated mice. Similar results were reported in *A*. *macrocarpa* [11].

Reduction in rectal body temperature is among the general features of *Plasmodium berghei*-infected mice. Measuring the rectal temperature of mice is used to predict the effectiveness of the tested plant against the parasite [37]. In this study, 400 mg/kg dose of the leaf latex of the plant was demonstrated a significant protective effect against reduction in body temperature as compared to negative control. Overall, this activity might probably indicate that the latex might prevent some pathological processes of malaria that cause reduction in body temperature and metabolic rates [27].

PCV is measured to evaluate the effectiveness of a plant in preventing hemolysis due to malaria infection [32]. The underlying cause of anemia includes the following mechanisms: the clearance and/or destruction of infected RBC, the clearance of uninfected RBC, and erythropoietic suppression and dyserythropoiesis [32]. The latex *of A*. *weloensis* prevented hemolysis of red blood cells in a dose-dependent manner. This could be due to the antimalarial activity of the traditional plant and as a result of sustaining the availability of new RBCs produced in the bone marrow. The current finding was in line with other reports on medicinal plants used to treat malaria, such as *Clerodendrum myricoides* leaves *and Dodonaea angustifolia* seeds [24, 38].

The mechanism of action of *A*. *weloensis* is not yet known. However, the existing literatures have shown that the possible antimalarial activity of the plant might be through antioxidation and free-radical scavenging, immunomodulatory, intercalation in deoxyribonucleic acid (DNA), inhibition of protein synthesis, and interference with enterocytes' invasion [26, 31]. Therefore, it is possible that the antiplasmodial activity exhibited by *A*. *weloensis* could have been as a result of the abovementioned ways or by yet a different unknown mechanism.

## 5. Conclusions

The acute toxicity test conducted on the leaf latex of *A*. *weloensis* confirmed the safety of the plant up to a dose of 2000 mg/kg. Furthermore, the findings of the present study indicated that the leaf latex of the plant possessed promising antimalarial activity. The antimalarial activity of the plant might be related to the presence of terpenoids, flavonoids, anthraquinones, glycosides, saponins, and tannins which might act single or in combination against *Plasmodium berghei* infection. Hence, the findings of this study genuinely support the claimed traditional use of *A*. *weloensis* for management of malaria. Hence, the plant might contain potential lead compounds for the development of novel antimalarial drugs.

## Figures and Tables

**Table 1:** Preliminary phytochemical screening of the leaf latex of *Aloe weloensis*.

Phytochemicals	Leaf latex of *Aloe weloensis*
Alkaloids	−
Anthraquinones	+
Flavonoids	+
Glycoside	+
Saponins	+
Tannins	+
Terpenoids	+

+ = presence; − = absence.

**Table 2:** Effect of *Aloe weloensis* on the parasitemia level, percentage suppression, and survival time of *Plasmodium berghei-*infected mice in the 4-day suppressive test.

Group	Dose (mg/kg)	Parasitemia level	(%) Suppression	Survival days
NC	10	8.71 ± 0.47	00.00	7.80 ± 0.37
*AW100*	100	7.57 ± 0.18^*∗*^	13.04^*∗*^	11.60 ± 0.51
AW200	200	5.06 ± 0.14^*∗∗*^	41.87^*∗∗*^	15.20 ± 0.86^*∗∗*^
AW400	400	2.89 ± 0. 11^*∗∗*^	66.84^*∗∗*^	20.00 ± 1.64^*∗∗*^
CQ25	25	0^*∗∗*^	100^*∗∗*^	>25^*∗∗*^

Notes: values are presented as Mean ± SEM; *n* = 5. ^*∗*^Values are significantly different (*p* < 0.05) as compared to negative control. ^*∗∗*^Values are more significantly different (*p* < 0.01) as compared to negative control. Abbreviations: AW, *Aloe weloensis*; CQ, chloroquine; NC, negative control; SEM, standard error of mean.

**Table 3:** Effect of *Aloe weloensis* on the temperature, weight, and packed cell volume of *Plasmodium berghei-*infected mice in the 4-day suppressive test.

Group	Dose (mg/kg)	BWt D_0_	BWt D_4_	Temp D_0_	Temp D_4_	PCV D_0_	PCV D_4_
NC	10 ml	28.80 ± 0.37	27.760 ± 0.23	37.12 ± 0.16	35.64 ± 0.36	49.60 ± 1.36	40.60 ± 1.81
AW100	100	28.00 ± 0.71	30.00 ± 0.37^*∗*^	37.34 ± 0.16	36.10 ± 0.27	48.00 ± 0.71	45.00 ± 0.95
AW200	200	26.80 ± 0.37	30.60 ± 1.52^*∗*^	36.58 ± 0.13	36.36 ± 0.31	48.80 ± 0.73	46.00 ± 0.63^*∗*^
AW400	400	28.60 ± 0.51	31.80 ± 0.68^*∗∗*^	36.54 ± 0.18	37.02 ± 0.07^*∗∗*^	49.00 ± 0.56	47.60 ± 1.08^*∗∗*^
CQ25	25	26.60 ± 0.51	32.60 ± 0.73^*∗∗*^	36.72 ± 0.12	37.12 ± 0.10^*∗∗*^	47.20 ± 0.37	48.80 ± 0.80^*∗∗*^

Data were expressed as mean ± SEM (*n* = 5); D_0_: before treatment; D_4_: after completing treatment. ^*∗*^Values are significantly different (*p* < 0.05) as compared to negative control. ^*∗∗*^Values are more significantly different (*p* < 0.01) as compared to negative control. Abbreviations: AW, *Aloe weloensis*; BWt, body weight (g); CQ, chloroquine; NC, negative control; PCV, packed cell volume; Temp, temperature; SEM, standard error of mean.

## Data Availability

Data can be obtained from the corresponding author on reasonable request.
